# Intrinsic Class D β-Lactamases of *Clostridium difficile*

**DOI:** 10.1128/mBio.01803-18

**Published:** 2018-12-18

**Authors:** Marta Toth, Nichole K. Stewart, Clyde Smith, Sergei B. Vakulenko

**Affiliations:** a Department of Chemistry and Biochemistry, University of Notre Dame, Notre Dame, Indiana, USA; b Stanford Synchrotron Radiation Lightsource, Stanford University, Menlo Park, California, USA; Louis Stokes Veterans Affairs Medical Center

**Keywords:** *Clostridium difficile*, antibiotic resistance, beta-lactamases

## Abstract

Clostridium difficile is the causative agent of the deadly C. difficile infection. Resistance of the pathogen to β-lactam antibiotics plays a major role in the development of the disease, but the mechanism of resistance is currently unknown. We discovered that C. difficile encodes class D β-lactamases, i.e., CDDs, which are intrinsic to this species. We studied two CDD enzymes, CDD-1 and CDD-2, and showed that they display broad-spectrum, high catalytic efficiency against various β-lactam antibiotics, including penicillins and expanded-spectrum cephalosporins. We demonstrated that the *cdd* genes are poorly expressed under the control of their own promoters and contribute only partially to the observed resistance to β-lactams. However, when the *cdd1* gene was expressed under the control of efficient promoters in the antibiotic-sensitive Clostridium cochlearium strain, it produced high-level resistance to β-lactams. Taken together, the results determined in this work demonstrate the existence in C. difficile of intrinsic class D β-lactamases which constitute a reservoir of highly potent enzymes capable of conferring broad-spectrum, clinically relevant levels of resistance to β-lactam antibiotics. This discovery is a significant contribution to elucidation of the mechanism(s) of resistance of the clinically important pathogen C. difficile to β-lactam antibiotics.

## Abstract (precis)

C. difficile is a spore-forming anaerobic bacterium which causes infection of the large intestine with high mortality rates. The C. difficile infection is difficult to prevent and treat, as the pathogen is resistant to many antimicrobial agents. Prolonged use of β-lactam antibiotics for treatment of various infectious diseases triggers the infection, as these drugs suppress the abundance of protective gut bacteria, allowing the resistant C. difficile bacteria to multiply. While resistance of C. difficile to β-lactam antibiotics plays the major role in the development of the disease, the mechanism of resistance is unknown. The significance of our research is in the discovery in C. difficile of β-lactamases, enzymes that destroy β-lactam antibiotics. These findings ultimately can help to combat deadly C. difficile infections.

## INTRODUCTION

Clostridium difficile infection is a life-threatening disease that imposes a significant burden on health care. In 2011, the estimated number of C. difficile infections in the United States exceeded 450,000, resulting in 29,300 deaths (1) and in associated excess health care costs of $4.8 billion for acute care facilities alone (2 – 4). The number of incidents of C. difficile infections in both hospitals and the outpatient setting is steadily rising, with an average annual rate of 3.3% in the United States (5 – 8). As a result, the sum of charges for C. difficile infection-associated hospitalizations increased from $20.1 billion in 2005 to $31.4 billion in 2014 (8). Antimicrobial therapy plays a crucial role in the development of C. difficile infection (9 – 11) due to the ability of many antibiotics to suppress the abundance of the gut microbiota that protects the host from invasion by pathogenic bacteria (12 – 14). Resistance of C. difficile to antibiotics is an important contributor to the emergence of the disease. C. difficile is notorious for its resistance to antimicrobial agents and is listed by the Centers for Disease Control and Prevention as the pathogen that causes the highest antibiotic resistance threat in the United States (15). While several classes of antimicrobial agents have been implicated in outbreaks of C. difficile infection, β-lactam antibiotics are recognized as major causative agents of the disease (16 – 18). β-Lactams differ significantly in their levels of activity against C. difficile (19, 20), and those levels correlate with their ability to trigger the infection. β-Lactams with low potency against C. difficile and high activity against the protective gut microbiota represent the major risk factor (21 – 24). Although resistance of C. difficile to β-lactams is recognized as a leading contributor to the development of C. difficile infection, the underlying mechanisms of resistance are currently unknown (19, 20).

## RESULTS

### C. difficile ATCC 43255 and C. difficile 630Δerm are resistant to β-lactam antibiotics.

While published data cumulatively indicate that C. difficile is generally resistant to many β-lactams (19, 20), there have been relatively few studies on the susceptibility of the pathogen to these antimicrobial agents. To initiate studies of the mechanism(s) of resistance of C. difficile to β-lactam antibiotics, we evaluated MIC values of various β-lactam antibiotics against two clinically important clostridial isolates, C. difficile ATCC 43255 and C. difficile 630Δerm (25 – 27), and showed that the two isolates exhibit very similar broad antibiotic resistance profiles (Table 1). They were highly resistant to the expanded-spectrum cephalosporins cefotaxime, ceftriaxone, ceftazidime, and cefepime (MICs, 64 to 256 µg/ml), the cephamycin cephalosporin cefoxitin (MIC, 128 µg/ml), and the monobactam aztreonam (MIC, 2,048 µg/ml). The MICs of the penicillins ampicillin, penicillin G, and oxacillin, of the carbapenems imipenem and meropenem, and of the cephalosporin cephalothin were significantly lower. The MICs of oxacillin, cephalothin, and meropenem were 2-to-8-fold higher for C. difficile 630Δerm.

**TABLE 1:** MICs of β-lactam antibiotics against clostridial isolates

Antibiotic	MIC (µg/ml)								
	*C. difficile* ATCC 43255	*C. difficile* 630Δerm			*C. cochlearium* ATCC 17787				
	*cdd1*	*cdd2*	*cdd2* intron	Δ*cdd2*	Parental strain	*cdd1	*cdd2	P*thl* *cdd1*	P*fdx* *cdd1*
Ampicillin	4	4	1	1	0.03	0.25	0.25	32	16
Penicillin G	4	4	1	1	0.015	0.25	0.25	32	16
Oxacillin	8	64	64	64	0.25	2	1	64	16
Cephalothin	16	32	32	32	0.12	0.5	0.25	32	16
Cefotaxime	128	128	128	128	1	2	2	128	64
Ceftriaxone	64	64	32	32	1	2	2	256	128
Cefoxitin	128	128	128	128	0.25	0.25	0.25	0.5	0.5
Ceftazidime	256	256	64	64	32	32	32	1,024	512
Cefepime	64	64	32	32	16	16	16	256	128
Aztreonam	2,048	2,048	2,048	2,048	512	512	512	512	256
Imipenem	4	4	4	4	0.03	0.06	0.06	0.12	0.12
Meropenem	2	4	4	4	0.007	0.015	0.015	0.03	0.03

### Genomes of C. difficile ATCC 43255 and C. difficile 630Δerm encode class D β-lactamases.

Gram-positive bacteria manifest resistance to β-lactam antibiotics via two major mechanisms: production of antibiotic-degrading enzymes, namely, β-lactamases, and/or production of antibiotic-resistant targets, namely, penicillin-binding proteins (PBPs). Four molecular classes of β-lactamases (classes A through D) are widely disseminated in Gram-negative bacteria, while for a long time, only class A and B enzymes were known in Gram-positive microorganisms. Recently, we demonstrated that class D enzymes are also widely spread in the *Clostridiaceae*, *Bacillaceae*, and *Eubacteriaceae* families of phylum *Firmicutes* of Gram-positive bacteria (28).

**FIG 1:**
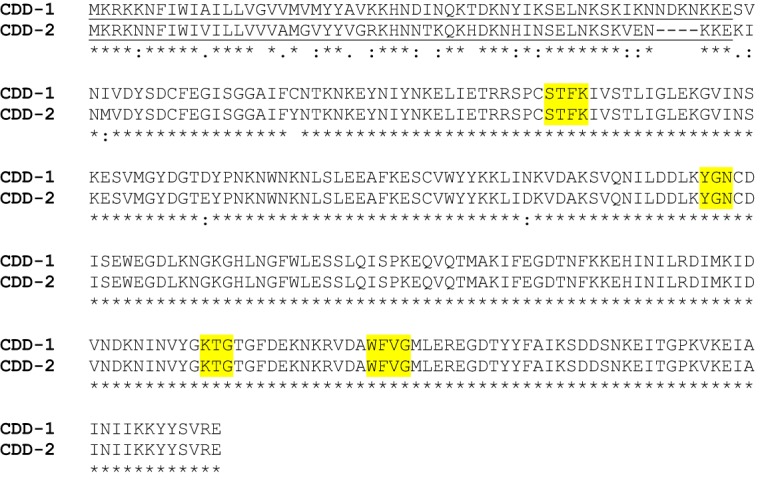
Amino acid sequence alignment and genomic regions of CDD-1 and CDD-2 β-lactamases. Sequence motifs conserved in class D β-lactamases are highlighted in yellow. Putative leader sequences for the CDD-1 and CDD-2 β-lactamases are underlined.

In our effort to get insights into the mechanism of resistance of C. difficile to β-lactam antibiotics, we analyzed genomes of two strains, C. difficile ATCC 43255 and C. difficile 630Δerm, and found that each contains only one open-reading frame annotated as a putative β-lactamase gene. The enzyme from C. difficile ATCC 43255 is 312 amino acid residues long, while that from C. difficile 630Δerm is slightly shorter (Fig. 1). Both harbor conserved amino acid sequence motifs (SXXK, YGN, KTG, and WXXG) characteristic of class D β-lactamases. We named the enzyme from C. difficile ATCC 43255 CDD-1 (an abbreviation from Clostridium difficile class D β-lactamase) and named the enzyme from C. difficile 630Δerm CDD-2.

### CDD β-lactamases are intrinsic to C. difficile.

To evaluate how widely CDD-type β-lactamases are spread among C. difficile, we analyzed around 800 completed genomic sequences of C. difficile and found that the vast majority of them harbor a single chromosomal gene for the CDD-type enzyme, showing that these enzymes are intrinsic to the pathogen. To appreciate the diversity of CDD-type β-lactamases on the amino acid sequence level, we utilized BLAST software from the NCBI for the search of homologous proteins using the sequence of the mature CDD-1 enzyme as the template. Results of this study showed that class D β-lactamases from C. difficile strains isolated from different parts of the world are very closely related, with >97% of them sharing at least 95% amino acid sequence identity.

To identify the most common substitutions in CDD β-lactamases, we analyzed the reported amino acid sequences that were identical in at least five different C. difficile strains. We found 477 such strains and subdivided their enzymes into two subgroups, the CDD-1-type and CDD-2-type subgroups (Table 2). We identified the CDD-1-type enzymes in 47 strains. Of those, 41 strains encoded the CDD-1 β-lactamase, while the 6 remaining strains encoded the CDD-1 mutant (CDD-1-M1) with a single C22Y amino acid substitution. This substitution is also present in enzymes of all other 430 strains encoding the CDD-2-type β-lactamases. Among the strains harboring genes for the CDD-2-type enzymes, the vast majority (312) encoded the CDD-2 β-lactamase. All remaining strains encoded seven CDD-2-like enzymes that have from one to three amino acid substitutions compared to the amino acid sequence of the CDD-2 β-lactamase.

**TABLE 2:** Most common amino acid substitutions in CDD β-lactamases

Enzyme^*a*^	Amino acid substitutions											
CDD-1 (41)^*b*^	**S2**	**V3**	**I5**	**G14**	**C22**	**D74**	**N103**	**S109**	**D165**	**R177**	**R205**	**L214**
CDD-1-M1 (6)					Y							
CDD-2 (312)	K	I	M		Y	E	D					
CDD-2-M1 (22)	K	I	M		Y	E	D				C	
CDD-2-M2 (13)	K	I	M		Y	E	D					I
CDD-2-M3 (11)	K	I	M		Y	E	D		Y			
CDD-2-M4 (24)	K	I	M		Y	E			Y			
CDD-2-M5 (9)	K	I	M		Y	E						
CDD-2-M6 (16)	K	I	M	D	Y	E		Y				
CDD-2-M7 (23)	K	I			Y	N				K		

a The number of isolates with the given amino acid substitutions is indicated in parentheses. CDD-2-M1 through CDD-2-M7 are mutants of CDD-1 and CDD-2 β-lactamases.

b The amino acid sequence of CDD-1, which was used as the reference sequence for comparison, is indicated in bold. The amino acid numbering indicates the position of each amino acid in the mature enzyme.

Our analysis also demonstrated that beyond the C. difficile species, the amino acid sequence similarity of CDD-1 to currently known class D enzymes is below 55%. These data indicated that CDD β-lactamases have failed to spread to other bacteria and that they represent a unique family of closely related enzymes that are native to C. difficile.

### C. difficile ATCC 43255 and C. difficile 630Δerm produce active CDD β-lactamases.

**FIG 2:**
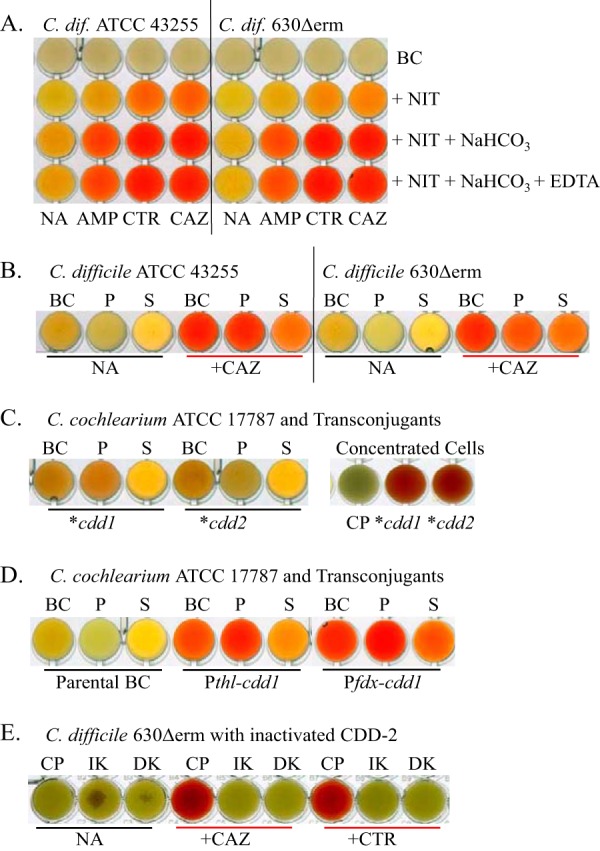
Evaluation of β-lactamase activity. (A) Induction of β-lactamase activity in C. difficile (*C. dif*) ATCC 43255 and C. difficile 630Δerm by three representative antibiotics. Their activity increases in the presence of NaHCO_3_ and is not inhibited by EDTA. (B) Distribution of β-lactamase between the bacterial cells and growth medium. (C) Expression of CDD-1 and CDD-2 enzymes from their own promoters in C. cochlearium ATCC 17787. The right panel shows 5-fold-concentrated cell pellets. (D) Expression of CDD-1 from two functional promoters in C. cochlearium ATCC 17787. (E) Loss of CDD-2 β-lactamase activity in C. difficile 630Δerm knockouts. Activity was measured in 5-fold-concentrated cell pellets. Abbreviations: BC, bacterial culture; NIT, nitrocefin; NA, no antibiotic; AMP, ampicillin; CTR, ceftriaxone; CAZ, ceftazidime; P, pellet; S, supernatant; CP, control pellet of the parental strain; IK, insertional knockout; DK, deletion knockout.

To assess whether C. difficile ATCC 43255 and C. difficile 630Δerm produce active β-lactamases, we utilized a nitrocefin test. We observed a slight change in nitrocefin color in wells with both strains grown in the presence of antibiotics but not in their absence (Fig. 2A, second row). We then investigated whether the observed β-lactamase activity was due to production of the class D CDD-1 and CDD-2 enzymes or if β-lactamases of other classes are involved. Unlike serine β-lactamases of classes A and C, class D enzymes require posttranslational carboxylation of their active-site lysine for catalysis (29). This lysine may undergo decarboxylation under experimental conditions, resulting in a precipitous decrease in the catalytic activity of the enzyme. The activity can be restored by addition of a CO_2_ source such as sodium bicarbonate. We monitored hydrolysis of nitrocefin in the presence of 50 mM sodium bicarbonate. We observed a significant change in color when bacteria were grown in the presence of each of the β-lactams that we tested (except for cefoxitin), while the color of antibiotic-free medium changed only slightly (Fig. 2A, third row). These data indicated that both C. difficile ATCC 43255 and C. difficile 630Δerm produce an inducible class D β-lactamase.

We also evaluated whether metallo β-lactamases contribute to the observed change in the nitrocefin color. The presence of EDTA, a chelating agent that strongly inhibits the activity of class B metalloenzymes, did not prevent or delay the nitrocefin reaction, which excludes the possibility of a contribution of metallo β-lactamases to nitrocefin hydrolysis (Fig. 2A, fourth row).

In Gram-positive bacteria, β-lactamases can be anchored to the bacterial cell wall and/or excreted to the milieu. We investigated whether the CDD-1 and CDD-2 enzymes are secreted or attached to the cell wall. We pelleted bacteria by centrifugation, saved the growth medium, and resuspended the pellets in a phosphate buffer. The nitrocefin color change in C. difficile ATCC 43255 was mostly associated with bacterial cell pellets, while the C. difficile 630Δerm color changes were similar in the cell pellets and supernatant (Fig. 2B). These data showed that more CDD β-lactamase is cell wall associated in C. difficile ATCC 43255 than in C. difficile 630Δerm. Cell wall association also has been demonstrated for other classes of β-lactamases from Gram-positive and Gram-negative bacteria, where the enzymes are often anchored to the membrane (30 – 33).

### The CDD-1 β-lactamase displays broad-spectrum activity.

To evaluate the substrate profile of CDD enzymes, we purified the CDD-1 β-lactamase to homogeneity and undertook kinetic studies. CDD-1 exhibited high catalytic efficiency for the three penicillins (*k*_cat_/*K_m_*, 1.2 × 10^6^ to >1.8 × 10^6^ M^−1^ s^−1^; Table 3) and for five of the six cephalosporins (*k*_cat_/*K_m_*, 1.9 × 10^5^ to 1.6 × 10^6^ M^−1^ s^−1^), with the exception of cefoxitin. The catalytic efficiency determined for the monobactam aztreonam (*k*_cat_/*K_m_*, 5.0 × 10^5^ M^−1^ s^−1^) was similar to that determined for the penicillins and cephalosporins, while that determined for the carbapenems imipenem and meropenem was 2 orders of magnitude lower. CDD-1 had high apparent affinity levels (*K_m_*, <1 to 2.7 µM) and relatively low turnover numbers (*k*_cat_, 1.8 to 3.2 s^−1^) for all penicillins tested. For the cephalosporins ceftriaxone, cefotaxime, and cefepime, turnover numbers were 4-to-8-fold higher and the apparent affinity decreased by up to 23-fold compared to penicillins. The steady-state kinetic parameters for aztreonam were similar to those for expanded-spectrum cephalosporins, while very low turnover rates corresponding to *k*_cat_ values of 0.044 and 0.005 s^−1^ were observed for imipenem and meropenem, respectively. Our kinetic studies demonstrated that CDD-1 is an efficient broad-spectrum β-lactamase. We also determined the steady-state kinetic parameters for the CDD-2 enzyme with several β-lactam antibiotics (ampicillin, oxacillin, cefotaxime, and ceftriaxone) and found they were almost identical to those of CDD-1 (data not shown).

**TABLE 3:** Steady-state kinetic parameters of the CDD-1 β-lactamase

Antibiotic	*k*_cat_ (s^−1^)	*K_m_* (µM)	*k*_cat_/*K_m_* (M^−1^ s^−1^)
Ampicillin	3.2 ± 0.1	2.7 ± 0.7	(1.2 ± 0.3) × 10^6^
Penicillin G	1.8 ± 0.1	<1	>(1.8 ± 0.1) × 10^6^
Oxacillin	2.9 ± 0.1	1.6 ± 0.3	(1.8 ± 0.3) × 10^6^
Cephalothin	0.88 ± 0.02	0.8 ± 0.2	(1.2 ± 0.2) × 10^6^
Cefoxitin	0.009 ± 0.001	40 ± 3	(2.3 ± 0.3) × 10^2^
Ceftazidime	21 ± 1	110 ± 10	(1.9 ± 0.2) × 10^5^
Cefepime	10.5 ± 0.2	23 ± 1	(4.5 ± 0.2) × 10^5^
Ceftriaxone	12.0 ± 0.4	7.6 ± 0.9	(1.6 ± 0.2) × 10^6^
Cefotaxime	12.0 ± 0.3	7.3 ± 0.7	(1.6 ± 0.2) × 10^6^
Aztreonam	20 ± 1	40 ± 3	(5.0 ± 0.5) × 10^5^
Imipenem	0.044 ± 0.001	4.7 ± 0.4	(9.4 ± 0.8) × 10^3^
Meropenem	0.005 ± 0.001	<4	>(1.3 ± 0.3) × 10^3^
Nitrocefin	40 ± 1	5.8 ± 0.8	(6.9 ± 1) × 10^6^

### The genes for CDD-1 and CDD-2 β-lactamases express poorly under the control of their own promoters.

**FIG 3:**
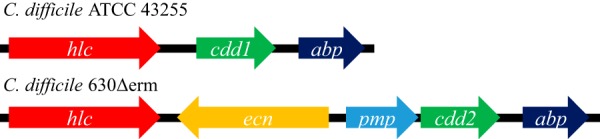
Schematic representation of regions surrounding the genes for CDD-1 and CDD-2 β-lactamases. The open reading frames are shown as arrows and intergenic regions as black lines. In strain ATCC 43255 (top), the gene for the CDD-1 β-lactamase (*cdd1*) is flanked by an ATP-dependent helicase (*hlc*) upstream and by an ABC transporter-coupled two-component system ATP-binding protein (*abp*) downstream. In strain 630 (bottom), two additional open reading frames are present between the gene for the CDD-2 β-lactamase (*cdd2*) and the *hlc* gene (an excinuclease ABC subunit A paralog of unknown function [*ecn*] and a putative membrane protein [*pmp*]). As a result, the intergenic region between the *cdd* gene and the upstream gene changed from 540 bp for *cdd1* to 27 bp for *cdd2*.

To test the expression levels of the *cdd1* and *cdd2* genes under the control of their own promoters, we analyzed the genetic composition of their upstream regions. The *cdd1* gene is flanked upstream by the gene for an ATP-dependent DNA helicase (Fig. 3). There is an insertion of two genes between *cdd*2 and the helicase gene in C. difficile 630Δerm; thus, the β-lactamase neighbors a putative membrane protein. Hence, intergenic regions upstream of the *cdd1* and *cdd*2 genes differ in length and nucleotide sequence. To establish whether there are functional promoters in close proximity to the *cdd1* and *cdd2* genes, we cloned those genes along with their 182-bp and 177-bp upstream regions, respectively, into the pMLT83151 vector, resulting in pMTL83151::*cdd1 and pMTL83151::*cdd2 constructs. As C. difficile is intrinsically resistant to expanded-spectrum cephalosporins, we expressed the CDD enzymes in a heterologous clostridial host, Clostridium cochlearium ATCC 17787, which we have shown is highly sensitive to most β-lactam antibiotics tested (Table 1) and is amenable to genetic manipulations.

We then introduced the pMTL83151::*cdd1 and pMTL83151::*cdd2 constructs in C. cochlearium ATCC 17787, where they were stably maintained. Expression of the CDD-1 and CDD-2 enzymes in the new host was not inducible by β-lactam antibiotics (data not shown). The change in nitrocefin color was slow with bacterial culture and was more intense with cell pellet than with the supernatant (Fig. 2C). The color change was more apparent when the cells were concentrated 5-fold. These data indicate that the CDD-1 and CDD-2 β-lactamases are poorly expressed from their own promoters in C. cochlearium ATCC 17787. The resulting low MIC values for the majority of β-lactam antibiotics tested support this assumption (Table 1). The largest effect was observed for penicillins, whose MIC values increased 8-fold to 16-fold to 0.25 to 2.0 µg/ml. The MIC values for cephalothin, cefotaxime, ceftriaxone, imipenem, and meropenem were increased 2-fold, while the MIC values of other β-lactams remained unchanged.

### CDD-1 β-lactamase produces high-level resistance to β-lactam antibiotics when expressed from efficient promoters.

Next, we investigated whether CDD β-lactamases are capable of producing high-level resistance to β-lactam antibiotics when expressed from known functional clostridial promoters. To accomplish this goal, we placed the gene for the CDD-1 β-lactamase encoding the full-length enzyme under the control of the P*thl* and P*fdx* promoters, which are often utilized for expression of clostridial genes in various clostridial species (34, 35). The resulting constructs were cloned into shuttle vectors to produce the pMTL83122::*cdd1*-P*thl* and pMTL83123::*cdd1*-P*fdx* vectors, which were introduced into C. cochlearium ATCC 17787. The nitrocefin color change seen with these constructs was significantly more intense than what we observed with CDD-1 expressed under the control of its own promoter (Fig. 2D and C). As in the case of C. difficile ATCC 43255, hydrolysis of nitrocefin was more efficient in cell pellets of C. cochlearium than in the supernatants, an indication that the two bacterial species process the CDD-1 β-lactamase in the same way (Fig. 2D).

Expression of CDD-1 from the P*thl* and P*fdx* promoters (P*thl cdd1* and P*fdx cdd1*) rendered C. cochlearium highly resistant to the majority of β-lactam antibiotics tested (Table 1). Overall, the P*thl* promoter provided 2-fold-higher levels of resistance. P*thl cdd1* increased the MICs of ampicillin, penicillin G, and oxacillin 256-fold to 2,048-fold (to 32 to 64 µg/ml). It also significantly increased the MICs of the majority of cephalosporins tested. The parental C. cochlearium ATCC 17787 strain is highly sensitive to cephalosporins cephalothin, cefotaxime, ceftriaxone, and cefoxitin. P*thl cdd1* increased the MIC values of the first three of these drugs 128-fold to 256-fold. Noticeably, the MICs of cefoxitin remained practically unchanged, an indication that this antibiotic is not a substrate for the β-lactamase. The two remaining cephalosporins, ceftazidime and cefepime, had much higher MICs against the parental C. cochlearium ATCC 17787 strain. Expression of the CDD-1 β-lactamase from the P*thl* promoter further increased the MICs of these antibiotics to 1,024 and 256 µg/ml, respectively. Expression of CDD-1 failed to further increase the high MICs of aztreonam. The MICs of meropenem and imipenem increased 4-fold but remained well below clinically significant levels.

### Inactivation of CDD-2 β-lactamase decreases resistance of C. difficile to β-lactam antibiotics.

To further evaluate the contribution of clostridial β-lactamases to the observed resistance to β-lactam antibiotics, we attempted to inactivate the genes for the CDD-1 and CDD-2 β-lactamases. Creating gene knockouts in C. difficile is challenging, as some C. difficile strains are minimally amenable to genetic manipulations, while the majority are not (36, 37). To inactivate the *cdd1* and *cdd2* genes, we utilized two recently developed methodologies. One of the two, represented by the ClosTron system, utilizes a group II intron to create insertions into the gene of interest to cause its inactivation (38). The other employs a two-step allele-exchange methodology (36) to generate targeted deletions of genetic material. Our numerous attempts to knock out the *cdd1* gene failed due to the extreme instability, in C. difficile ATCC 43255, of the vectors used for generation of knockouts. We succeeded, however, in creating the *cdd2* gene knockout in C. difficile 630Δerm using both approaches. Inactivation of the gene abolished the β-lactamase activity as confirmed by the nitrocefin test (Fig. 2E). The MICs of β-lactams against C. difficile 630Δerm strain harboring either of the two generated *cdd2* gene knockouts (*cdd2* intron and Δ*cdd2*) were identical (Table 1). We observed a 4-fold decrease in the MIC values for ampicillin, penicillin G, and ceftazidime and a 2-fold decrease for ceftriaxone and cefepime, while the MICs of other antibiotics remained unchanged. This correlates with poor expression of the gene under the control of its own promoter, which we also observed in C. cochlearium. These data demonstrated that while C. difficile 630Δerm encodes a class D β-lactamase that has high catalytic activity against various β-lactam substrates, the enzyme is poorly expressed in this strain and responsible only partially for its resistance to these antibiotics. Results of our studies also indicate that an additional, as-yet-unidentified mechanism in C. difficile 630Δerm contributes significantly to the observed high-level resistance to β-lactams.

We then aimed to reintroduce the *cdd2* gene into the C. difficile 630Δerm strain harboring the Δ*cdd2* gene knockout and to evaluate the effect on the produced resistance. For this purpose, we used the pMTL83151::*cdd2 construct, where the gene is expressed under the control of its own promoter, and also constructed the pMTL83122::*cdd2*-P*thl* plasmid, where the *cdd2* gene is expressed under the control of the efficient P*thl* promoter. We then measured the MICs of the two β-lactams, ampicillin and ceftriaxone, against the resulting transconjugants. For the transconjugant harboring the pMTL83151::*cdd2 plasmid, we observed only a 2-fold increase in the MICs for one of antibiotics, ampicillin, while for the transconjugant carrying the pMTL83122::*cdd2*-P*thl* construct, the MIC of ampicillin increased 16-fold and that of ceftriaxone 2-fold (data not shown). These data clearly show that the *cdd2* gene is expressed more efficiently under the control of the P*thl* promoter than under that of its own promoter, as was also the case with the *cdd1* gene in C. cochlearium. The lower magnitude of the increase in antibiotic resistance in C. difficile could have resulted from both its significant level of residual β-lactam resistance and the high degree of instability of the constructs that we observed in this host.

## DISCUSSION

In our efforts to elucidate the mechanism of resistance of C. difficile to β-lactam antibiotics, we discovered that this pathogen encodes intrinsic class D β-lactamases which share a high level of identity of their amino acid sequences. Antibiotic susceptibility testing of two clostridial isolates, C. difficile ATCC 43255 and C. difficile 630Δerm, showed that they are resistant to expanded-spectrum cephalosporins and the cephamycin cefoxitin and are highly resistant to the monobactam aztreonam but are sensitive to both penicillins and carbapenems. Our kinetic studies of CDD β-lactamases demonstrated that both CDD-1 and CDD-2 possess high catalytic activity against a wide spectrum of β-lactam antibiotics. Comparison of the antibiotic resistance and catalytic profiles of CDD-1 revealed some noticeable discrepancies between them (Tables 1 and 3). While the pathogen was highly resistant to cefoxitin, CDD-1 had very low catalytic efficiency against this substrate, which indicates that the high MIC of cefoxitin likely resulted from low affinity of the drug to its target PBP. The opposite trend was observed for ampicillin, penicillin G, oxacillin, and cephalothin. Although CDD-1 has high catalytic efficiency against these substrates, C. difficile ATCC 43255 is relatively sensitive to these antibiotics. Such sensitivity could result from poor expression of the CDD-1 β-lactamase.

To validate this assumption, we knocked out the *cdd2* gene and observed only moderate decreases in the MICs of β-lactams. Subsequent reintroduction of the *cdd2* gene under the control of its own and the strong P*thl* promoters confirmed that the β-lactamase is indeed poorly expressed under the control of its own promoter. In addition, we expressed the genes for the CDD-1 and CDD-2 β-lactamases in the highly β-lactam-sensitive C. cochlearium ATCC 17787 strain and found that both enzymes were also expressed poorly from their own promoters in this bacterium.

Our study also demonstrated that the catalytic efficiency or substrate profile or both of the CDD enzymes are unique among class D β-lactamases. Although CDD-1 has a substrate profile similar to that of the only other characterized class D β-lactamase from Gram-positive bacteria, BPU-1, it has superior (up to 200-fold-higher) catalytic efficiency against expanded-spectrum cephalosporins and aztreonam (28). While the substrate profile of CDD-1 is much broader than that of mutants of OXA-2 and OXA-10 β-lactamases from Pseudomonas aeruginosa (39, 40), it is more similar to those of some mutant class D β-lactamases from Acinetobacter baumannii (41 – 44). However, as with BPU-1, the catalytic activity of CDD-1 is much higher. Our analysis demonstrates that, among characterized class D β-lactamases, CDD-1 overall displays the broadest spectrum and the highest level of activity against expanded-spectrum cephalosporins and aztreonam. These results strongly indicate that active site of CDD-1 has a unique architecture that evolved to accommodate and efficiently hydrolyze a wide spectrum of β-lactam substrates.

Combined, our experiments showed that C. difficile strains produce intrinsic broad-spectrum CDD β-lactamases. In the two studied strains, the enzymes are poorly expressed and responsible only partially for the observed β-lactam resistance. This implies that these strains also employ an additional, non-CDD β-lactamase-mediated mechanism(s) of resistance. Importantly, our studies demonstrated that the CDD β-lactamases are very potent enzymes, and when one of them, CDD-1, was supplied with functional promoters, it produced high-level, broad-spectrum resistance to β-lactams. Further experiments are warranted to determine whether CDD β-lactamases with efficient promoters are circulating in any of the multiple clinical C. difficile isolates. In fact, acquisition of strong promoters has already been well documented with class D β-lactamase genes of Acinetobacter baumannii. Originally, these genes were poorly expressed and were of no clinical concern. However, over time, they acquired stronger promoters supplied by insertion sequences, which rendered A. baumannii highly resistant to carbapenem antibiotics. As C. difficile also harbors multiple mobile genetic elements, which constitute up to 11% of its genome (45), the acquisition of strong promoters by the *cdd* genes is highly likely. More-efficient promoters would provide even higher levels and broader spectra of resistance to β-lactam antibiotics. Indeed, clinical C. difficile isolates with such high levels of resistance to β-lactams have already been reported (20, 46, 47). Regardless, these intrinsic enzymes pose a serious potential threat, as they are fully capable of producing a high level of resistance to clinically important antibiotics, which would further complicate the already difficult tasks of preventing and treating C. difficile infection.

## MATERIALS AND METHODS

### Bacterial strains.

E. coli DH10B (New England Biolabs) was used in cloning experiments. E. coli CA434 (CHAIN Biotech, United Kingdom) was used as the donor strain for mobilization of shuttle vectors into clostridial strains. C. difficile 630Δerm (CHAIN Biotech, United Kingdom) and C. difficile ATCC 43255 are known toxigenic isolates, widely used to study various aspects of C. difficile infection. C. cochlearium ATCC 17787 was used for expression of *cdd* genes. All clostridial isolates were grown under anaerobic conditions at 37°C.

### Antibiotic susceptibility testing.

MICs of β-lactam antibiotics against clostridial strains were determined according to Clinical and Laboratory Standards Institute guidelines (M11-A8) (48). Briefly, the broth microdilution method was used with 96-well plates. Serial dilutions of antibiotics were performed in supplemented Brucella medium, which subsequently was inoculated with the final bacterial inoculum of 10^6^ CFU/ml. Plates were incubated for 44 to 48 h at 37°C in an anaerobic box prior to recording results. All MIC experiments were performed at least in triplicate.

### Identification of β-lactamases in C. difficile.

To identify β-lactamases in C. difficile ATCC 43255 and C. difficile 630Δerm, we analyzed their genomic sequences (available at https://www.ncbi.nlm.nih.gov). Visual inspection of the amino acid sequences of these enzymes was used to identify conserved motifs of the class D β-lactamases. Around 800 completed genomic sequences of C. difficile (available at www.patricbrc.org) were analyzed to appreciate the spread and diversity of CDD enzymes.

### Nitrocefin test.

To assess whether clostridia produced active β-lactamases, we utilized the chromogenic cephalosporin nitrocefin, which changes color from yellow to dark red upon hydrolysis. Overnight bacterial cultures were used for the analysis of bacterial cultures, pellets resuspended in phosphate buffer, and supernatants. Reactions performed with nitrocefin (200 µM) were monitored for up to 60 min. Sodium bicarbonate and EDTA were used at 50 mM and 10 mM, respectively, where indicated.

### Cloning procedures.

For expressing the CDD-1 β-lactamase in C. cochlearium ATCC 17787, we placed the full-length *cdd1* gene under the control of either the P*thl* promoter or P*fdx* promoter (34, 35) by cloning it between the NdeI and HindIII restriction sites of the pMTL83122 and pMTL83123 shuttle vectors (49) to generate pMTL83122::*cdd1* and pMTL83123::*cdd1*, respectively.

To evaluate whether there are functional promoters upstream of the *cdd1* and *cdd2* genes, we PCR amplified DNA fragments containing the entire genes and their 182-bp and 177-bp 5′ flanking regions, respectively. We subsequently cloned the amplified fragments between the BamHI and HindIII restriction sites of the pMTL83151 vector to generate pMTL83151::*cdd1 and pMTL83151::*cdd2, respectively.

To construct the *cdd1* and *cdd2* gene knockouts, we utilized two alternative methodologies. The ClosTron system achieves gene inactivation by the insertion of a group II intron (38), while a two-step allele exchange technique generates targeted deletions of genetic material (36). For the insertional mutagenesis, the introns were retargeted using an online tool (www.clostron.com), custom synthesized, and inserted into the pMTL007C-E5 vector (ATUM, CA), resulting in pMTL007C-E5::Cdi-*cdd1*-284a and pMTL007C-E5::Cdi-*cdd2*-272a. For the deletional inactivation of the *cdd1* and *cdd2* genes, we amplified regions upstream (left arm) and downstream (right arm) of the gene fragment to be deleted. The left arm included the first 30 bp of the genes and 759 bp of their upstream region. The right arm consisted of the 511 bp of the 3′ region of the *cdd* genes plus 560 bp downstream. Use of this construct is expected to create deletions of 398 bp and 386 bp in the *cdd1* and *cdd2* genes, respectively. The arms were ligated, and the product was cloned into the PmeI restriction site of the pMTL-SC7315 vector to result in pMTL-SC7315::Δ*cdd1* and pMTL-SC7315::Δ*cdd2*.

To produce a large amount of the CDD-1 β-lactamase, the gene for the mature enzyme was custom synthesized (GenScript) and cloned into the NdeI and HindIII sites of the pET-24a(+) expression vector (Novagen). The mature enzyme lacks the first 58 N-terminal amino acids, whose deletion is necessary for the optimal expression of the enzyme, as we have shown previously for the BPU-1 class D β-lactamase from another Gram-positive bacterium, Bacillus pumilus (28).

### Conjugation experiments.

Various shuttle vectors were delivered to clostridial cells using an established protocol (49). Briefly, 1 ml of overnight culture of donor E. coli CA434 culture harboring a shuttle vector was pelleted and resuspended in 200 µl of the appropriate C. difficile or C. cochlearium recipient strains. The bacterial suspension was spotted onto the surface of brain heart infusion-supplemented (BHIS) agar and incubated anaerobically overnight at 37°C. Next, grown cells were resuspended in 500 µl BHIS medium and spread on selective plates to recover transconjugants. For C. cochlearium, selection was modified by using 12 µg/ml aztreonam instead of cefoxitin. After 3 days of incubation and subsequent restreaking, individual colonies were grown in selective BHIS medium overnight. DNA was isolated using a DNeasy blood & tissue kit (Qiagen) and analyzed by PCR to identify transconjugants.

### Inactivation of *cdd1* and *cdd2* genes using ClosTron mutagenesis.

The mutagenesis procedure was performed as described earlier (37). Briefly, following introduction of pMTL007C-E5::Cdi-*cdd1*-284a and pMTL007C-E5::Cdi-*cdd2*-272a into C. difficile ATCC 43255 and C. difficile 630Δerm, respectively, the transconjugants were selected on BHIS agar supplemented with 20 µg/ml lincomycin and 250 µg/ml d-cycloserine overnight at 37°C. Next, 120 individual colonies were restreaked onto the same medium, and the resulting individual colonies were grown in BHIS agar supplemented with 20 µg/ml lincomycin for DNA isolation. Insertion of the intron into the *cdd* genes was verified by PCR and sequencing.

### Inactivation of *cdd1* and *cdd2* genes by using the two-step allele exchange procedure.

The protocol was used as previously described (36). pMTL-SC7315::Δ*cdd1* and pMTL-SC7315::Δ*cdd2* were introduced into C. difficile ATCC 43255 and C. difficile 630Δerm, respectively, by conjugation. The transconjugants were restreaked onto TCC plates (BHIS agar supplemented with 15 µg/ml thiamphenicol, 10 µg/ml cefoxitine, and 250 µg/ml d-cycloserine), and the DNA from larger colonies was isolated and checked by PCR for integration of the plasmid into the chromosome. An integrant clone was then restreaked onto nonselective BHIS agar plates, and the cells were harvested in 0.5 ml phosphate-buffered saline (PBS) after 3 to 4 days of incubation and spread onto C. difficile minimal medium (CDMM) plates supplemented with 75 µg/ml 5-fluorocytosine after serial 10^2^ to 10^5^ dilutions. Plates with individual colonies were replica plated onto BHIS agar with and without 15 µg/ml thiamphenicol to identify clones that lost the plasmid. Deletion of the *cdd* genes was confirmed by PCR and sequencing.

### Protein expression and purification.

For expression of the CDD-1 enzyme, BL21(DE3) cells harboring the plasmid with the *cdd* gene were grown at 37°C with shaking to an optical density at 600 nm (OD_600_) of 0.6. Expression was induced with 0.5 mM isopropyl β-d-thiogalactoside (IPTG), and the cells were grown overnight at 22°C. The cells were harvested by centrifugation, resuspended in 25 mM HEPES (pH 7.0), 1 mM EDTA, and 0.2 mM dithiothreitol (DTT), and disrupted by sonication. Subsequently, the lysate was centrifuged at 32,000 rpm at 4°C and the supernatant was loaded onto a DEAE anion exchange column (Bio-Rad) preequilibrated with the same buffer. The protein was eluted with a 0 to 250 mM NaCl gradient. The enzyme was concentrated using a Centricon Plus 70 concentrator (Millipore), and dialyzed against 25 mM HEPES (pH 7.0) using a 10-kDa-molecular-weight-cutoff (MWCO) membrane (Spectrum).

### Enzyme kinetics.

All data were collected on a Cary 60 spectrophotometer (Agilent). Reaction conditions were identical to those previously reported for class D β-lactamases (50). Observed rates were calculated from the linear phase of each reaction and fitted to the Michaelis-Menten equation using GraphPad Prism 5.04 for Windows (GraphPad Software, CA) to determine the steady-state parameters *k*_cat_ and *K_m_*. Standard deviations were calculated using the same software.
